# Lack of Association between Intact/Deletion Polymorphisms of the *APOBEC3B* Gene and HIV-1 Risk

**DOI:** 10.1371/journal.pone.0092861

**Published:** 2014-03-25

**Authors:** Mayumi Imahashi, Taisuke Izumi, Dai Watanabe, Junji Imamura, Kazuhiro Matsuoka, Hirotaka Ode, Takashi Masaoka, Kei Sato, Noriyo Kaneko, Seiichi Ichikawa, Yoshio Koyanagi, Akifumi Takaori-Kondo, Makoto Utsumi, Yoshiyuki Yokomaku, Takuma Shirasaka, Wataru Sugiura, Yasumasa Iwatani, Tomoki Naoe

**Affiliations:** 1 Department of Infectious Diseases and Immunology, Clinical Research Center, National Hospital Organization Nagoya Medical Center, Nagoya, Japan; 2 Department of Hematology and Oncology, Graduate School of Medicine, Nagoya University, Nagoya, Japan; 3 Department of Hematology and Oncology, Graduate School of Medicine, Kyoto University, Kyoto, Japan; 4 Japanese Foundation for AIDS Prevention, Chiyoda-ku, Tokyo, Japan; 5 Clinical Research Center, National Hospital Organization Osaka Medical Center, Osaka Japan; 6 Center for Human Retrovirus Research, Institute for Virus Research, Kyoto University, Kyoto, Japan; 7 Department of International Health Nursing, Graduate School of Nursing, Nagoya City University, Nagoya, Japan; 8 Department of AIDS Research, Graduate School of Medicine, Nagoya University, Nagoya, Japan; University Hospital Zurich, Switzerland

## Abstract

**Objective:**

The human APOBEC3 family of proteins potently restricts HIV-1 replication *APOBEC3B*, one of the family genes, is frequently deleted in human populations. Two previous studies reached inconsistent conclusions regarding the effects of *APOBEC3B* loss on HIV-1 acquisition and pathogenesis. Therefore, it was necessary to verify the effects of APOBEC3B on HIV-1 infection *in vivo*.

**Methods:**

Intact (I) and deletion (D) polymorphisms of *APOBEC3B* were analyzed using PCR. The syphilis, HBV and HCV infection rates, as well as CD4^+^ T cell counts and viral loads were compared among three *APOBEC3B* genotype groups (I/I, D/I, and D/D). HIV-1 replication kinetics was assayed *in vitro* using primary cells derived from PBMCs.

**Results:**

A total of 248 HIV-1-infected Japanese men who have sex with men (MSM) patients and 207 uninfected Japanese MSM were enrolled in this study. The genotype analysis revealed no significant differences between the *APOBEC3B* genotype ratios of the infected and the uninfected cohorts (p = 0.66). In addition, HIV-1 disease progression parameters were not associated with the *APOBEC3B* genotype. Furthermore, the PBMCs from D/D and I/I subjects exhibited comparable HIV-1 susceptibility.

**Conclusion:**

Our analysis of a population-based matched cohort suggests that the antiviral mechanism of APOBEC3B plays only a negligible role in eliminating HIV-1 *in vivo*.

## Introduction

Human APOBEC3 proteins are cellular cytidine deaminases that play crucial roles in the inhibition of retroviral replication, including that of HIV-1 [1]–[3]. The molecular mechanisms underlying APOBEC3-mediated HIV-1 restriction are primarily dependent on the editing [1], [2] and/or non-editing activities [4], [5] of these enzymes. The family of genes encoding the seven APOBEC3 proteins (APOBEC3A, B, C, DE, F, G, and H) is positioned in a tandem array on human chromosome 22 [6]. HIV-1 produces an accessory protein, Vif, that invalidates the antiviral functions of the APOBEC3 proteins by mediating the ubiquitination-proteasomal degradation of APOBEC3 in virus-producing cells [7]. APOBEC3C, DE, F, G, and H (haplotype II) are vulnerable to HIV-1 Vif-mediated degradation, whereas APOBEC3A and B are resistant [8]–[12].

Among the members of the APOBEC3 family, APOBEC3G has been consistently shown to possess powerful anti-HIV-1 activity in cell-based systems [1], [2], and this protein may affect the pathogenesis of HIV-1 infection *in vivo* [13]–[19]. However, there is little consensus regarding the degree to which the other APOBEC3 family members, especially APOBEC3B, are able to restrict HIV-1 replication *in vitro* and *in vivo*. The anti-HIV-1 activity of APOBEC3B is undetectable when this gene is stably expressed in a human T cell line [20] and is detected only weakly after the transient transfection of HEK 293T or HeLa cells [20]–[22]. Because these findings have varied according to the experimental conditions employed, there is a fundamental question whether the expression of human APOBEC3B, DE, and F plays a critical role in HIV-1 restriction *in vivo*. The potential role of APOBEC3B in modulating HIV-1 replication *in vivo* is of particular interest because this protein is resistant to HIV-1 Vif-mediated degradation [20], [22]–[24].

A polymorphic deletion of a 29.5-kb segment between *APOBEC3A* exon 5 and *APOBEC3B* exon 8 has been identified in human populations; this polymorphism causes the loss of the entire APOBEC3B coding region [25]. A particularly high frequency of *APOBEC3B* deletion has been found among Asians [25]. According to Kidd et al., the deletion allele is rare in Africans (1%) and Europeans (6%), more common in East Asians (36%) and Amerindians (58%), and almost fixed in Oceanians (93%) [25].

Two independent groups have reported contrasting findings concerning the effects of the *APOBEC3B* gene deletion on HIV-1 acquisition and disease progression [26], [27]. An et al. determined that the deletion allele genotype correlated with a higher risk of HIV-1 infection, whereas a study conducted by Itaya et al. concluded that the deletion polymorphism had no effect on HIV-1 acquisition and the rate of disease progression to AIDS. An et al. included 4 patients with homozygous deletions of *APOBEC3B* in their HIV-1-seropositive cohorts of 656 European and 296 African-American individuals but no homozygotes for the deletion in their seronegative groups, which prevented a proper evaluation of the impact of the deletion polymorphism on HIV-1 acquisition and pathogenesis [26]. In contrast, the study conducted by Itaya et al. in Japan utilized inappropriate enrollment [27], because the enrolled patients were all hemophiliacs who had survived HIV-1 infection for at least 10 years prior to the study and the information for individuals who had progressed to AIDS and death before the enrollment date was excluded.

To examine the impact of the *APOBEC3B* deletion polymorphism on HIV-1 infection risk *in vivo*, this study enrolled a matched cohort in Japan and investigated the impact of *APOBEC3B* gene intact/deletion polymorphisms on HIV-1 susceptibility and pathogenesis. In addition, we analyzed the effects of different *APOBEC3B* genotypes on HIV-1 replication kinetics *in vitro*.

## Materials and Methods

### Sample Collection

A total of 248 Japanese HIV-1-positive men who have sex with men (MSM) who were patients at Nagoya Medical Center (n = 203) and Osaka Medical Center (n = 45) were enrolled in this study from November 2011 to February 2013. The control group comprised 207 Japanese HIV-1-negative MSM who were recruited at the Nagoya Lesbian & Gay Revolution Plus (NLGR+) festival in June 2012. The study protocol was approved by the ethics committees of Nagoya Medical Center (registration number 2011-430) and Osaka Medical Center. Written informed consent was obtained from all the participants. The control subjects recruited at the NLGR+ festival provided anonymous consent. To collect information regarding their sex, nationality, age, and sexuality, anonymous questionnaires collated with linked numbers were obtained.

### Genotyping

The *APOBEC3B* intact (I) and deletion (D) alleles were genotyped using a previously reported polymerase chain reaction (PCR) method [26] with slight modifications. Of note, the “intact (I)” in this study is used for the “insertion” that originally reported by Kidd et al. [25]. Briefly, the primer sets for amplifying the Deletion and Insertion 2 fragments were the same as those previously described [26], although one additional set of primers for the Insertion 1 fragement was replaced by the two following oligonucleotide primers: Insertion3_F: 5′-GAGTGGAAGCGCCTCCTC-3′ and Insertion3_R: 5′-CTCCTGGCCAGCCTAGC-3′. The QIAamp DNA Blood Mini Kit (Qiagen, Valencia, USA) was used according to the manufacturer’s protocol to extract genomic DNA from whole blood (patients) or from buccal mucosa (controls).

### Analysis of Viral Replication Capacity and Infectivity

Peripheral blood mononuclear cells (PBMCs) were isolated from fresh blood samples from different HIV-1-negative donors with the I/I and D/D *APOBEC3B* genotypes (n = 5 for each) using Ficoll-Hypaque density gradient centrifugation (Pharmacia, Uppsala, Sweden). The PBMCs were then subjected to negative selection with the MACS CD4 T Cell Isolation Kit (Miltenyi Biotec, Cologne, Germany) to purify primary CD4^+^ T cells. The cells were activated with 1 μg/ml of phytohemagglutinin (PHA) (Pharmacia) for 72 hours, infected with HIV-1 NL4-3 for 24 hours with a multiplicity of infection (MOI) of 0.01, washed twice, and maintained in RPMI-1640 medium with 20% fetal bovine serum (FBS), penicillin (50 U/ml)/streptomycin (50 μg/ml) (Invitrogen, Carlsbad, USA), and 20 U/ml interleukin-2 (IL-2) (Roche Applied Science, Mannheim, Germany). The culture supernatants were assayed for the p24 antigen using the HIV-1 p24 Antigen Assay Kit (Coulter Corporation, Fullerton, USA) on the day of infection and on days 2, 4, 6, 8, 10, and 13 after infection. To analyze the viral infectivity of the infected PBMCs, culture supernatants were harvested six days post-infection and inoculated into TZM-bl cells [29] in black 96-well plates. The viral infectivity was assessed 48 hours post-infection by detecting β-galactosidase activity using the Galacto-Star System (Applied Biosystems, Foster City, USA).

### Quantification of APOBEC3 mRNA

To analyze the mRNA expression levels of members of the APOBEC3 family, unstimulated CD4^+^ cells from three different genotyped subjects were prepared for RNA isolation. The induction rates of mRNA transcription for APOBEC3A or APOBEC3G were analyzed using monocyte-derived macrophages (MDMs). Briefly, monocytes were isolated from PBMCs from each genotyped healthy donor using CD14 MicroBeads (Miltenyi Biotec). The enriched CD14^+^ cells were plated at a cell density of 1×10^6^/ml in 12-well plates in RPMI-1640 medium (Sigma, St. Louis, USA) with penicillin (50 U/ml)/streptomycin (50 μg/ml) for three hours, followed by the addition of 10% FBS and 10 ng/ml macrophage colony stimulating factor (M-CSF) (Peprotech, Rocky Hill, USA). Adherent cells were cultured for eight days to facilitate their differentiation into MDMs. Differentiated MDMs either received no stimulation or were stimulated with 100 U/ml of recombinant human interferon (IFN)-α (Sigma) for six hours and were then lysed for RNA isolation. As previously described [14], [30], total RNA isolated using the QIAamp RNA Blood Mini Kit (Qiagen) was used to synthesize cDNA with the SuperScript III First-Strand Synthesis System (Invitrogen) using random hexamers. The cDNA levels were quantified using real-time PCR in a Thermal Cycler Dice Real Time System (TP800) (Takara Bio, Shiga, Japan). The real-time PCR was employed to analyze the levels of APOBEC3, β-actin, and GAPDH mRNA, and the assays were performed according to the manufacturer’s protocol using SYBR Premix DimerEraser (Takara Bio). The primer sets for the real-time PCR were purchased from FASMAC Co., Ltd. (Atsugi, Japan) and the oligonucleotide sequences are shown in Table S1. The gene expression levels were calculated using the ΔΔCt (Ct; cycle threshold) and are presented as the ratio of APOBEC3 mRNA to β-actin or GAPDH mRNA.

### Statistical Analysis

The relationships between *APOBEC3B* genotype and baseline characteristics were assessed using the Fisher exact test for categorical variables. The Mann-Whitney *U*-test was used for continuous variables. All the statistical analyses were performed with the statistical software EZR (Saitama Medical Center, Jichi Medical University), which is a graphical user interface for R (The R Foundation for Statistical Computing, version 2.13.0). More specifically, this software is a version of R commander (version 1.6–3) modified to add statistical functions that are frequently used in biostatistics [31]. All the p values were two-tailed. The effects of *APOBEC3B* gene deletion on the disease progression of HIV-1 were evaluated based on the CD4+ T cell counts and log10 HIV-1 viral load (RNA copy number/ml) at more than two time points before the start of antiretroviral therapy (ART). Patients whose CD4^+^ T cell counts and HIV-1 viral loads were measured at fewer than two time points were excluded from the statistical analyses of these factors. Other related infectious diseases were identified in the patients using the following definitions. If the rapid plasma reagin test and/or the *Treponema pallidum* latex agglutination (TPHA) test were positive, the patient was considered positive for syphilis. Patients were considered hepatitis B virus (HBV)-positive if either hepatitis B surface antigen (HBsAg) or hepatitis B core antibody (HBcAb) was present. In addition, patients were considered hepatitis C virus (HCV) carriers if they tested positive for HCV antibodies.

## Results

### *APOBEC3B* Genotype Frequencies in the Cohorts

The demographics of the HIV-1-positive and HIV-1-negative cohorts are shown in Table 1. A total of 248 HIV-1-infected Japanese MSM patients and 207 uninfected Japanese MSM were enrolled and analyzed in this study. To conduct a matched cohort study, all the participants were recruited from Nagoya and Osaka in the central area of Japan. First, a comparative analysis of the *APOBEC3B* genotype among the participants indicated that there were no significant differences in *APOBEC3B* genotype frequency between the HIV-1-positive (D/D 7.7%, I/D 44.0%, and I/I 48.4%) and HIV-1-negative (D/D 8.7%, I/D 39.6%, and I/I 51.7%) cohorts (p = 0.66) (Table 1). A comparison of the distributions of the *APOBEC3B* deletion allele in the HIV-1-positive and HIV-1-negative cohorts revealed that the D allele occurred in the HIV-1-positive (29.6%) and HIV-1-negative subjects (28.5%) at comparable rates (p = 0.71). We also analyzed the cDNA sequences of *APOBEC3B* I allele isolated from the Japanese healthy donors with the I/I or I/D genotypes (Table S1). There was one variant (rs#2076109): K62 (allele frequency, or AF = 0.4) (E62 as the reference) although we could not detect any other variants changing the amino acid sequences within the 15 alleles. According to the 1000 Genome database, the variant (AF = 0.373) appears globally distributed but not limited in Japan or Asia. In addition, we tested the antiviral effect of APOBEC3B E62 and the variant with an overexpression system using 293T cells (Figure S1). The results demonstrated that the E62 variant had equivalent antiviral activity to APOBEC3B K62 *in vitro*. These data suggest that the I alleles in our Japanese cohorts are not strongly biased in terms of genetic and functional features.

**Table 1:** *APOBEC3B* genotype frequency in HIV-1-positive patients and HIV-1-negative controls.

	HIV-1		
	Negative (%)	Positive (%)	p^a^
Genotype			
D/D	18/207 (8.7)	19/248 (7.7)	0.66
I/D	82/207 (39.6)	109/248 (44.0)	
I/I	107/207 (51.7)	120/248 (48.4)	
Allele			
D	118/414 (28.5)	147/496 (29.6)	0.71
I	296/414 (71.5)	349/496 (70.4)	

a Determined using the Fischer exact test.

Next, we analyzed the HIV-1-positive individuals for the prevalence of HBV, HCV, and syphilis, as well as for HIV-1 disease progression at a minimum of two time points before the commencement of ART. The prevalence of each infectious disease is presented in Table 2. The frequencies of the three *APOBEC3B* genotypes (D/D 7.6%, I/D 45.4%, and I/I 47.0%) among the 132 HBV-positive patients were not significantly different from those of the 94 HBV-negative individuals (D/D 9.6%, I/D 41.5%, and I/I 48.9%) (p = 0.69). In addition, the *APOBEC3B* genotype distributions did not differ significantly between the HCV-positive and HCV-negative patients (p = 1.00) or between the syphilis-positive and syphilis-negative patients (p = 0.62) (Table 2).

**Table 2:** *APOBEC3B* genotype frequency and clinical parameters in HIV-1-positive patients.

	APOBEC3B genotype			
	D/D (%)	I/D (%)	I/I (%)	p^a^
HBV				
Positive	10/132 (7.6)	60/132 (45.4)	62/132 (47.0)	0.69
Negative	9/94 (9.6)	39/94 (41.5)	46/94 (48.9)	
Unknown	0/22 (0)	10/22 (45.5)	12/22 (54.5)	
HCV				
Positive	0/7 (0)	3/7 (42.9)	4/7 (57.1)	1.00
Negative	19/241(7.9)	106/241 (44.0)	116/241 (48.1)	
Syphilis				
Positive	9/116 (7.8)	47/116 (40.5)	60/116 (51.7)	0.62
Negative	10/131 (7.6)	61/131 (46.6)	60/131 (45.8)	
Unknown	0/1 (0)	1/1 (100)	0/1(0)	

a Determined using the Fischer exact test.

**Figure 1:**
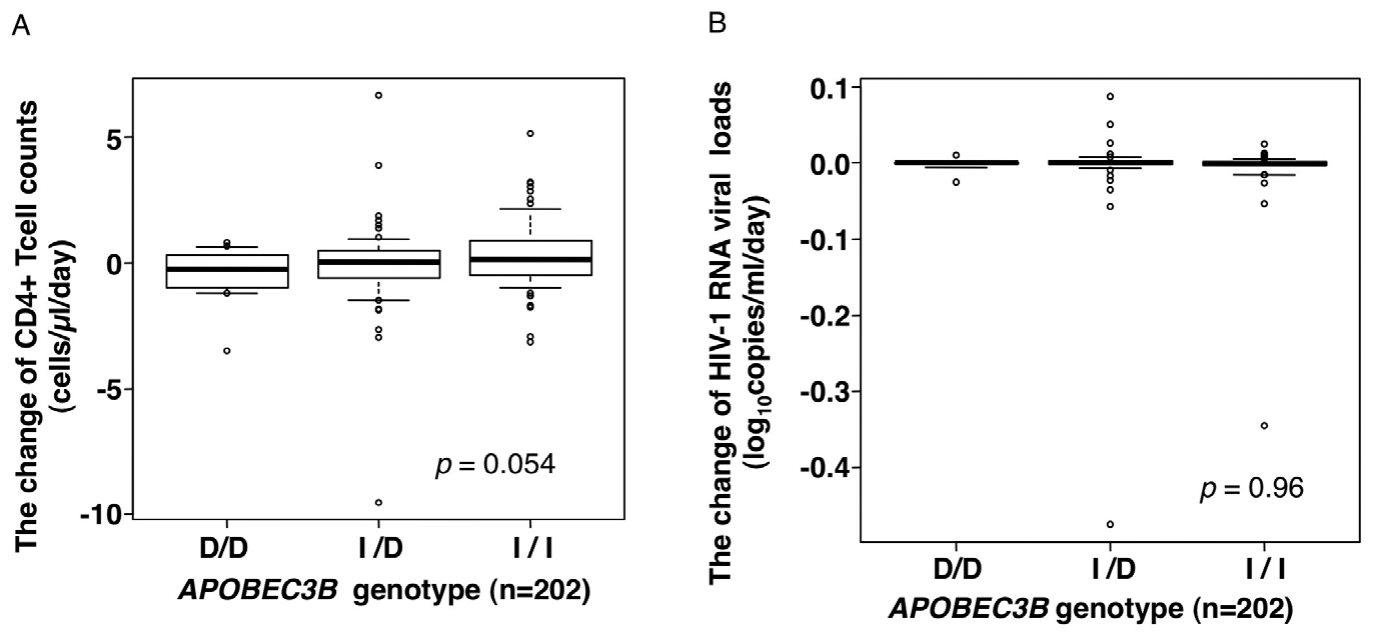
Analysis of effects of genotype on parameters of HIV disease progression in the HIV-1-infected cohort. (A) Changes in CD4^+^ T cell counts (cells/μl/day) (n = 202). (B) Changes in HIV-1 RNA levels (log_10_ copies/ml/day) in plasma (n = 202). The box plots show data between the 25th and 75th percentiles with central horizontal lines representing the median, and with whiskers showing the 10th and 90th percentiles. The open circles represent outliers with data >1.5-fold of the interquartile range. All the p values were determined using the Kruskal-Wallis test.

We also assessed the rates of both CD4^+^ T cell decline and plasma viral load increase at different time points after the first patient visit to the hospital prior to ART treatment. As shown in Figure 1, the changes in the CD4^+^ T cell counts (cells/μl/day) and viral loads (log_10_ copies/ml/day) did not differ significantly according to *APOBEC3B* genotype (CD4: p = 0.054; viral load: p = 0.96). The data from the 46 patients (D/D 6.5%, I/D 41.3%, and I/I 52.2%) who began ART before the second measurement of the CD4^+^ T cells and viral loads were excluded from the analysis. Of these 46 patients, 32 (D/D 3.1%, I/D 50.0%, and I/I 46.9%) began ART shortly after their first hospital visit due to AIDS onset; this decision was based on the domestic clinical guidelines of the Ministry of Health, Labor, and Welfare of Japan. There were no significant differences in the proportions of the *APOBEC3B* genotypes between the patients with CD4^+^ T cell count and viral load data from at least two time points and the 46 patients without complete data (p = 0.91). Detailed demographic information on the HIV-1 (+) patients is shown in Table 3. Moreover, we analyzed the non-ART periods from the first diagnosis through the ART introduction and set two groups: longer and shorter than median days from diagnosis to ART. As the genotype frequencies were compared (Table 4), the results showed no significant difference in the *APOBEC3B* genotype between the two groups (p = 0.96).

**Table 3:** Demographics of the cohorts.

	HIV-1 Negative(n = 207)	HIV-1 Positive(n = 248)
Age (years), median [IQR]^a^	33 [26]–[39]	40 [36–51]
Year of diagnosis, median [IQR] a	NA^b^	2008 [2005–2010]
ART naïve at entry, n (%)	NA^b^	20 (8%)
CD4^+^ cell count at entry (cells/mm^3^), median [IQR] a	NA^b^	451 [294–534]
HIV-1 viral load at entry (copies/mL), median [IQR] a	NA^b^	61 [<40–410]
History of AIDS, n (%)	NA^b^	32 (13%)
Days from diagnosis to entry, median [IQR] a	NA^b^	1470 [539–2256]
Observation periods for disease progression (days), median [IQR] a	NA^b^	56 [28–88]
Days from diagnosis to ART, median [IQR] a	NA^b^	88 [38–599]

a IQR denotes interquartile range.

b NA, Not applicable.

**Table 4:** *APOBEC3B* genotype frequency on the days from diagnosis to ART (n = 246).

		days from diagnosis to ART		
		88 days> (%)	88 days< (%)	p^a^
Genotype				
D/D		9 (3.7)	10 (4.1)	0.961
I/D		49 (19.9)	59 (24.0)	
I/I		57 (23.2)	62 (25.2)	

(Median days from diagnosis to ART = 88 days).

a Determined using the Fischer exact test.

The diagnosis date of 2 patients(each patient’s genotype is I/I and I/D, respectively.) are unknown.

Moreover, we performed deep sequencing of the HIV-1 proviral DNAs that were isolated from the I/I, I/D or D/D patients’ PBMCs, and then analyzed the hypermutation rates on APOBEC3-prefered dinucleotide sequences: GG>AG and GA>AA mutations. The results showed that the hypermutation frequencies vary among different individuals although the levels of GA>AA hypermutation relative to the GG>AG are comparable among the three *APOBEC3B* genotypes (Figure S2). The data suggest that the APOBEC3B is not likely a major contributor to introduce hypermutations on the proviral DNAs in HIV-1(+) patients’ PBMCs.

### The Effects of *APOBEC3B* Genotype on Other APOBEC3 Expression Profiles

**Figure 2:**
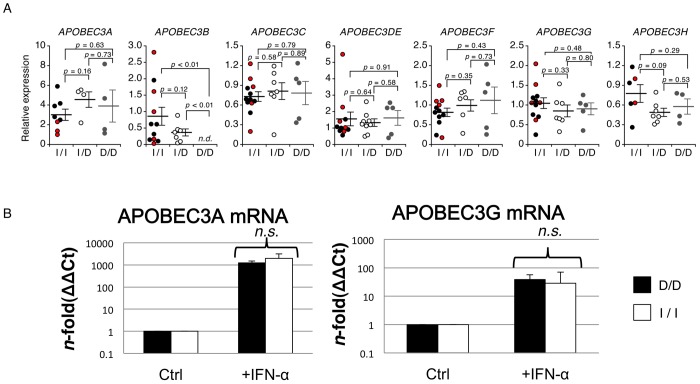
APOBEC3 mRNA expression levels depending on *APOBEC3B* genotype. (A) Comparison of mRNA expression levels of APOBEC3 in CD4^+^ cells isolated from intact (I/I), hemizygous (I/D) and deletion (D/D) individuals of healthy donors. The relative mRNA expression levels of APOBEC3A (I/I, n = 8; I/D, n = 4; D/D, n = 4), APOBEC3B (I/I, n = 11; I/D, n = 7; D/D, n = 5), APOBEC3C (I/I, n = 12; I/D, n = 7; D/D, n = 5), APOBEC3DE (I/I, n = 11; I/D, n = 9; D/D, n = 5), APOBEC3F (I/I, n = 12; I/D, n = 7; D/D, n = 5), APOBEC3G (I/I, n = 12; I/D, n = 7; D/D, n = 5), and APOBEC3H (I/I, n = 6; I/D, n = 7; D/D, n = 4) were determined using quantitative RT-PCR and were normalized to GAPDH. The red (I/I) or gray (D/D) dots represent the expression levels of donors whose PBMCs were used for the *in vitro* kinetics of HIV-1 replication and infectivity in Figure. 3. The p values were calculated using Welch’s *t*-test. The error bar represents the standard error of the mean (SEM). (B) APOBEC3A (A3A) and APOBEC3G (A3G) mRNA expression levels under basal conditions (Ctrl) and after stimulation with 100 U/ml (+IFN-α) of interferon (IFN)-α in CD14^+^ MDMs isolated from healthy control subjects. The black and white bars indicate D/D (n = 3) and I/I (n = 4) individuals, respectively. The *p* values were calculated with the Mann-Whitney *U*-test. The error bars represent the standard deviation. *n.d.*, not detected. Ct, cycle threshold. *n.s.*, not significant (*p* = 0.4 for both cases).

To assess whether the *APOBEC3B* gene deletion altered the expression of the other proximal *APOBEC3* genes, we compared mRNA expression profiles in fresh, unstimulated primary CD4^+^ cells of each *APOBEC3B* genotype: D/D, I/D, and I/I. As shown in Figure 2A, the mRNA expression levels of APOBEC3A, which is the *APOBEC3* family member located closest to the *APOBEC3B* gene, were not significantly different between the I/I and D/D genotype groups (p = 0.63), although the levels would likely vary considerably among individuals. As expected, APOBEC3B mRNA expression levels were not detected in the D/D subjects (Figure 2A). The APOBEC3B mRNA levels in the I/D subjects were somewhat lower than in the I/I subjects, although this difference was not statistically significant (Figure 2A, p = 0.12). Moreover, the relative levels of APOBEC3C, DE, F, G, and H mRNA were comparable among the I/I, I/D, and D/D subjects (Figure 2A). We also analyzed APOBEC3B mRNA levels in PBMCs isolated from healthy donors and HIV-1 seropositive patients with or without the ART. Similar to the pattern of APOBEC3B mRNA levels in the CD4+ T cells of three genotyped subjects (Figure 2A), the mRNA expression is slightly lower in the I/D genotyped PBMCs than in the I/I whereas no detectable level of APOBEC3B mRNA in the D/D PBMCs (Figure S3). The different expression levels between the I/D and I/I PBMCs were not statistically significant (Figure S3). Moreover, comparative analysis showed that the APOBEC3B mRNA level of each I/I or I/D genotype appears relatively higher in the HIV-1 (+) patients, regardless the ART-treatment, than in the uninfected donors. However, the difference was not statistically significant (Figure S3).

In the *APOBEC3B* D allele, the APOBEC3A mRNA contains a 3′-untranslated region of APOBEC3B’s and is subject to the upstream regulatory elements of the *APOBEC3A*. Thus, we further assessed whether the degrees to which APOBEC3A and APOBEC3G mRNA expression was stimulated by IFN-α in MDMs differed between the *APOBEC3B* I/I and D/D genotypes. The APOBEC3G mRNA expression was used as a control because the gene is distal to the *APOBEC3B* loci on the genome. As shown in Figure 2B, IFN-α stimulation resulted in APOBEC3A mRNA increases in the I/I and D/D MDMs of 1,999±1,190-fold and 1,251±264-fold, respectively. The APOBEC3G mRNA levels increased upon IFN-α stimulation by 28.6±41.8-fold (I/I) and 38.9±18.0-fold (D/D). A comparison of the mRNA expression magnitudes between the two homozygous *APOBEC3B* genotypes revealed no significant differences (p* = *0.4 and p* = *0.4 for APOBEC3A and APOBEC3G, respectively).

### The Effects of *APOBEC3B* Genotype on HIV-1 Susceptibility *in Vitro*

**Figure 3:**
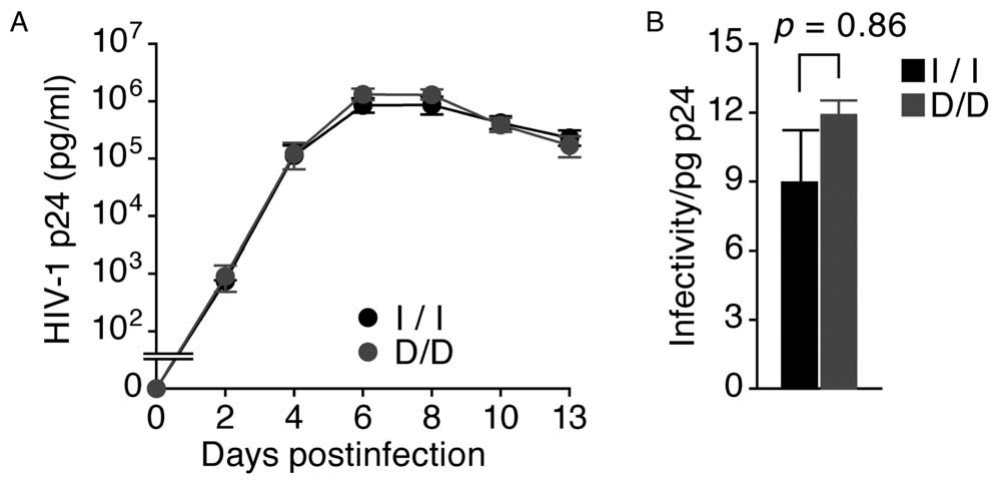
The kinetics and infectivity of HIV-1 depending on *APOBEC3B* genotype. (A) The kinetics of HIV-1 replication in PBMCs isolated from I/I (black dot) or D/D (gray dot) subjects (n = 5 each). (B) The infectivity values of virus-containing supernatants derived from I/I (black bar) and D/D (gray bar) PBMCs six days post-infection are provided relative to the values normalized with equal amounts of p24. The assay was performed using samples from three donors, and a representative result is shown. The p values were calculated using Welch’s *t*-test. The error bars represent the SEM.

We further analyzed the viral replication kinetics in primary PBMCs isolated from D/D or I/I donors. At an MOI of 0.01, the efficiency of HIV-1 replication was comparable between the D/D and I/I genotypes (Figure 3A). The p24 antigen levels in the culture supernatant from the I/I and D/D PBMCs were 8.7±3.0×10^5 ^pg/ml and 1.3±0.2×10^6^ pg/ml, respectively, on day 8 (p* = *0.31) and 8.4±0.2×10^5^ pg/ml and 1.3±0.3×10^6^ pg/ml, respectively, on day 6 (p* = *0.13). At the peak of infection (day 6), the virus-containing supernatants derived from D/D and I/I PBMCs exhibited comparable levels of infectivity (p* = *0.86) (Figure 3B). These data suggest that the different *APOBEC3B* deletion genotypes are not associated with significantly different levels of HIV-1 susceptibility *in vitro*.

## Discussion

There is only limited information about the roles played by APOBEC3 family members *in vivo*, with the exception of APOBEC3G. Previously, two independent groups reported conflicting conclusions regarding the impact of the *APOBEC3B* gene deletion on human HIV-1 infection *in vivo*, and this issue remains unclear [26], [27]. Therefore, to determine the effects of different *APOBEC3B* genotypes on HIV-1 infection *in vivo* and *in vitro*, we investigated the frequencies of intact and deletion polymorphisms of the *APOBEC3B* gene in a matched cohort in Japan.

The comparison of *APOBEC3B* genotypes in HIV-1-infected patients and HIV-1-negative controls revealed similar *APOBEC3B* genotype distributions in the two groups: D/D 7.7%, I/D 44.0%, and I/I 48.4% in the infected cohort versus D/D 8.7%, I/D 39.6%, and I/I 51.7% in the uninfected cohort (p = 0.66). In addition, no significant associations between the *APOBEC3B* genotype and the subclinical parameters of disease progression were observed among the HIV-1-positive patients. We also found no differences between the mRNA expression profiles of other APOBEC3 family members in PBMCs. Furthermore, the IFN-α-stimulated mRNA induction rates for APOBEC3A and APOBEC3G in MDMs did not differ between the D/D and I/I genotypes. Moreover, the HIV-1 susceptibility levels in PBMCs were comparable between the two genotypes. Considered together, our findings suggest that the loss of APOBEC3B is not significantly associated with HIV-1 acquisition and pathogenesis *in vivo* and with HIV-1 susceptibility *in vitro*, which fully supports the results of the cohort study conducted by Itaya et al [27].

There are two possible explanations for the lack of APOBEC3B involvement in HIV-1 restriction. First, the APOBEC3B protein cannot be incorporated into viral cores. Efficient HIV-1 restriction requires that APOBEC3 family proteins are packaged into virions through associations with viral and/or nonviral RNA [1], [2], [28]–[30] and that the proteins are localized to the plasma membrane in virus-producing cells [31]. APOBEC3G colocalizes with HIV-1 RNA and cellular RNA in P bodies [32] and are dispersed throughout the cytoplasm that facilitate interactions with HIV-1 Gag proteins and their incorporation into nascent virions [1], [2]. In contrast, APOBEC3B predominantly localizes to the nucleus [20], [21], [33], which may prevent its incorporation into virions.

The second possible explanation is that the low expression level of APOBEC3B in PBMCs [22], [34], [35] is insufficient to block HIV-1 replication, as shown in Figure 3. Similar to the HIV-1 results, overexpressed APOBEC3B potently suppresses HBV replication *in vitro* [36]. However, a study by Abe et al. on the frequency of the D/D genotype in HBV carriers demonstrated that the *APOBEC3B* gene deletion was not responsible for chronic HBV infection [37]. These data suggest that the high expression of APOBEC3B *in vitro* may produce exaggerated effects on both HIV-1 and HBV infection *in vitro*.

All the participants enrolled in this study were Japanese MSM, according to the information provided on anonymous questionnaires. Because approximately 80% of the HIV-1-positive patients in Japan are MSM [38], we investigated the effects of *APOBEC3B* deletion polymorphisms on this major mode of HIV-1 transmission rather than on the two other major modes (injection drug use and heterosexual intercourse). However, the effect of *APOBEC3B* genotype is less likely to be dependent on the mode of HIV-1 transmission because APOBEC3B mRNA expression in hematopoietic cells is lower and less tissue-specific than that of most of the other APOBEC3 family members [22], [34], [35].

In the D/D genotype, APOBEC3A mRNA expressed from the genome has a 3′-untranslated region corresponding to that of APOBEC3B. In addition, the genomic location of the APOBEC3G coding region is closer to the highly IFN-responsive transcriptional element of *APOBEC3A* in the D/D genome than to in the I/I. Therefore, we evaluated whether *APOBEC3B* gene deletion altered the IFN-stimulated gene induction of the other APOBEC3 family members. Our results suggest that the 29.5-kb genomic deletion of *APOBEC3B* does not significantly affect the expression profiles of the proximal *APOBEC3* family genes. Therefore, it is unlikely that the loss of the *APOBEC3B* gene in the D/D population leads to functional compensation via the mRNA expression modulation of the other APOBEC3 family members. Interestingly, Biasin et al. have demonstrated that increased levels of APOBEC3G mRNA in PBMCs, (primarily CD14^+^ MDMs) following exposure to IFN-α correlated with HIV-1 susceptibility both *in vivo* and *in vitro* [13]. Our results showed that the induction magnitude of APOBEC3G mRNA upon the IFN-α stimulation was similar between the I/I and D/D genotypes (Figure 2B). This suggests that different HIV-1 susceptibility observed by Basin et al. is unlikely linked to the *APOBEC3B* intact/deletion genotypes.

Recent studies of tumors such as breast cancers [39]–[41] and lymphomas [42] have shown that increased expression of APOBEC3B *in vivo* was linked to the chronic induction of mutations and/or instability in genomic DNA. We did not observe any significant diagnosable HIV-associated cancers in our short-term cohort study. It may be necessary to continue our prospective studies for a longer period. In addition, because other studies have suggested that *APOBEC3B* gene deficiency is associated with higher susceptibility to two other ancient pathogens, human T-cell leukemia virus type 1 [43], [44] and *Plasmodium falciparum* (the causative agent of malaria) [45], it would be beneficial to further investigate the correlations between *APOBEC3B* genotype and susceptibility to unknown pathogens.

## Conclusions

Our analysis of a population-based matched cohort provided important evidence that the loss of the *APOBEC3B* gene is not associated with risk of HIV-1 infection and disease progression. In addition, the *in vitro* kinetics of HIV-1 replication and the infectivity of the virus in PBMCs were comparable between the D/D and I/I subjects. These results suggest that the APOBEC3B antiviral mechanism plays only a negligible role in eliminating HIV-1 *in vivo*. This finding may explain why HIV-1 has not evolved a Vif-based strategy to counteract APOBEC3B restriction. Further analyses to explore the role(s) of APOBEC3B in human are also required in other cohorts with diverse genetic backgrounds in Asia.

## Supporting Information

Figure S1 **Overexpression of two APOBEC3B variants and the antiviral effect of the variants** ***in vitro*** **. (A)** A DNA fragment of the complete *APOBEC3B* open reading frame was amplified by RT-PCR from each RNA sample of healthy donors with APOBEC3B K62 (A3B K62) and E62 (A3B E62). Each of the fragment was replaced into the *APOBEC3G* gene position of the pcDNA A3G (Myc-His) WT (A3G WT) plasmid as previously described [8]. The primer sets for amplification of APOBEC3B cDNA were used as follows: the 1st PCR, 5′- gagcgggacagggacaagcg and 5′- aacccaggtctctgccttcc; the 2nd PCR, 5′- tcgagcggccgcatgaatccacagatcagaaatccg and 5′- cgatacaagcttgtttccctgattctggagaatggc. The resultant APOBEC3B expression plasmids, pcDNA APOBEC3B K62 and pcDNA APOBEC3B E62, contain a C-terminal MycHIS tag (consisting of Myc and hexa-histidine epitopes). The sequences of both the insert and the boundary regions for the APOBEC3B expression plasmids were verified by DNA sequencing. The expression or control (Vector) plasmids were transfected into human embryonic kidney cells (HEK 293T) by using FuGENE HD (Promega, Madison, USA). At 48 hr after transfection, cell lysates were prepared with Laemmli buffer containig 2.5% 2-Mercaptoethanol and analyzed by western blot. Protein bands were probed with anti-β-tubulin rabbit polyclonal antibody (1/2,500) (ab6046, Abcam, Cambridge, USA) or anti-His mAb (1/3,000) (D291-3, Medical & Biological Laboratories Co., Nagoya, Japan) as previously reported [8]. **(B)** The effect of two APOBEC3B variants on HIV-1 infectivity *in vitro* was analyzed. For virus production, 293T cells were cotransfected with 1 μg of pNL4-3 WT (HIV-1 WT) or pNL4-3*vif*(–) (HIV-1 *vif*(–)) plus 1 (black) or 0.1 (gray) μg of pcDNA APOBEC3B K62, pcDNA APOBEC3B E62, pcDNA 3.1 (–) (Vector), or pcDNA A3G (Myc-His) WT. Because it has been reported that the antiviral effect of APOBEC3B on HIV-1 *in vitro* can be observed when overexpressed in 293T cells but not T cell lines [20], we used 293T cells for the virus production. Virus infectivity was determined using TZM-bl cells [29]. Relative infectivity as relative light units (RLU) was calculated by normalizing for the amount of input CA, determined by p24 antigen ELISA (ZeptoMetrix, Buffalo, USA). Three independent experiments were performed. Results from one representative experiments are shown. A3G, APOBEC3G.
(TIF)

Figure S2 **Quantitative hypermutation analysis of APOBEC3-prefered dinucleotide motifs in the proviral DNA isolated from PBMCs of HIV-1 (+) patients. (A)** Genomic DNAs from patients’ PBMC (n = 4, for each *APOBEC3B* genotype I/I, I/D, and D/D) were extracted using the QIAamp DNA Blood Mini Kit. The proviral DNA fragments were prepared by nested PCR using the PrimeSTAR GXL DNA Polymerase (Takara Bio). For the first PCR, a 2,877-bp DNA fragment of *pol* (RT-IN) region (nt 2,388–5,264 according to the numbering positions of HXB2 strain, K03455) and a 1,095-bp fragment of *vif* region (nt 4,899–5,993) were independently amplified with 300 nM of each primer set: *pol*, DRRT1L (5′-atgatagggggaattggaggttt) and DRIN1R (5′-cctgtatgcagaccccaatatg); *vif*, DRVIF1F (5′-cgggtttattacagggacagcag) and DRVIF1R (5′-gctgtctccgcttcttcctgccat). For the nested PCR, a 2,735-bp (*pol*, nt 2,485–5,219) and an 859-bp (*vif*, nt 4,953–5,812) fragment were generated using primer sets, DRRT7L (5′-gacctacacctgtcaacataattgg)/DRRT7R (5′-cctagtgggatgtgtacttctgaactta) and DRVIF2F (5′-ctctggaaaggtgaaggggcagta)/DRVIF2R (5′-gaataatgcctattctgctatg), respectively. The resulting PCR products were purified with the QIAquick PCR Purification kit (Qiagen) and quantified with the Quant-iT dsDNA BR kit (Life Technologies). Paired-end DNA libraries were prepared using the Nextera DNA sample prep kit (Illumina, San Diego, USA) according to the manufacture’s protocol. The DNA libraries were sequenced on a MiSeq (Illumina) using the MiSeq reagent kit v2 to produce 250 bp ×2 paired-end reads. The reads generated by deep sequencing were mapped onto the reference sequence of HXB2 strain by BWA 0.7.3a program (http://bio-bwa.sourceforge.net). Then, sequences of a 150-base pairs-long region were extracted and the sequences containing bases with quality scores under 30 were omitted by our *in house* program. **(B)** Among the extracted sequences, the hypermutation types and the numbers of the dinucletitide sequences, GG>AG (red) and GA>AA (blue), were analyzed. In order to detect hypermutations, the unique sequences with >5-fold coverage depth were used. The frequency (%) of hypermutaion is shown as mutation rates per dinucleotide (GG or GA) sequence with two color-coded scales below. The positions of the hypermutations in each patient sample are represented based on the nucleotide position of HXB2 strain. Since no sequences at the 3′-end part of *vif* (5470–5619) in sample ID #15, 47 and 73 were mapped onto HXB2 (panel A), the hypermutation frequency in the portion (5,485–5,619) is not shown. **(C)** The cumulative histograms represent number of hypermuated positions (y-axis) for GG>AG (red) or GA>AA (blue) at the degree of hypermutation (%) (x-axis). Bars were denoted for every 10% of the frequency degree.
(PDF)

Figure S3 **Relative expression levels of APOBEC3B mRNA in three different** ***APOBEC3B*** **genotyped subjects of healthy donors or HIV-1-infected patients.** Total RNA samples were isolated from PBMCs of three different genotyped subjects, intact (I/I); hemizygous (I/D); and deletion (D/D) individuals, of healthy donors (Uninfected), or HIV-1 (+) patients before (Naïve) or after (Treated) cART. Each genotype includes 3 samples for each status. Relative APOBEC3B mRNA expression levels were determined by using RT-qPCR using the Thermal Cycler Dice Real Time System (TP800) (Takara Bio, Shiga, Japan). The qPCR cycle at which amplification was detectable above a background threshold (threshold cycle, or Ct) was calculated and normalized to β-Actin. The relative expression levels are presented as the ΔΔCt (*n*-fold) of APOBEC3B mRNA to β-actin mRNA. cDNA sytheisis and qPCR was performed in duplicate for each sample, and the mean values and standard deviations for each genotype group (n = 3) are shown. The p values were calculated using Kruskal-Wallis test. The error bar represents the standard deviation. *n.d.*, not detected.
(TIF)

Table S1 **Oligonucleotide primers used for real-time PCR of** ***APOBEC3*** **and control.** A real-time PCR assay was performed for *APOBEC3* and control genes (Gene symbol) using each of forward primer (S) and reverse primer (AS) sets.
(DOC)

Table S2 **APOBEC3B variations in the I/D and I/I genotyped healthy donors.** APOBEC3B cDNAs from I/D and I/I-genotyped healthy donors (n = 5 each) were amplified by the nested RT-PCR and cloned into pUC118 plasmids. The *APOBEC3B* cDNA sequences were determined by DNA sequencing. The individual APOBEC3B variants analyzed are shown. A3B, APOBEC3B.
(DOC)
