# Supplementary material for: Lack of Association between Intact/Deletion Polymorphisms of the APOBEC3B Gene and HIV-1 Risk
Source: PLoS One. 2014 Mar 25;9(3):e92861. doi: 10.1371/journal.pone.0092861 (PMC3965477; doi:10.1371/journal.pone.0092861)
Supplement: Figure S2 — Quantitative hypermutation analysis of APOBEC3-prefered dinucleotide motifs in the proviral DNA isolated from PBMCs of HIV-1 (+) patients. (A) Genomic DNAs from patients’ PBMC (n = 4, for each APOBEC3B genotype I/I, I/D, and D/D) were extracted using the QIAamp DNA Blood Mini Kit. The proviral DNA fragments were prepared by nested PCR using the PrimeSTAR GXL DNA Polymerase (Takara Bio). For the first PCR, a 2,877-bp DNA fragment of pol (RT-IN) region (nt 2,388–5,264 according to the numbering positions of HXB2 strain, K03455) and a 1,095-bp fragment of vif region (nt 4,899–5,993) were independently amplified with 300 nM of each primer set: pol, DRRT1L (5′-atgatagggggaattggaggttt) and DRIN1R (5′-cctgtatgcagaccccaatatg); vif, DRVIF1F (5′-cgggtttattacagggacagcag) and DRVIF1R (5′-gctgtctccgcttcttcctgccat). For the nested PCR, a 2,735-bp (pol, nt 2,485–5,219) and an 859-bp (vif, nt 4,953–5,812) fragment were generated using primer sets, DRRT7L (5′-gacctacacctgtcaacataattgg)/DRRT7R (5′-cctagtgggatgtgtacttctgaactta) and DRVIF2F (5′-ctctggaaaggtgaaggggcagta)/DRVIF2R (5′-gaataatgcctattctgctatg), respectively. The resulting PCR products were purified with the QIAquick PCR Purification kit (Qiagen) and quantified with the Quant-iT dsDNA BR kit (Life Technologies). Paired-end DNA libraries were prepared using the Nextera DNA sample prep kit (Illumina, San Diego, USA) according to the manufacture’s protocol. The DNA libraries were sequenced on a MiSeq (Illumina) using the MiSeq reagent kit v2 to produce 250 bp ×2 paired-end reads. The reads generated by deep sequencing were mapped onto the reference sequence of HXB2 strain by BWA 0.7.3a program (http://bio-bwa.sourceforge.net). Then, sequences of a 150-base pairs-long region were extracted and the sequences containing bases with quality scores under 30 were omitted by our in house program. (B) Among the extracted sequences, the hypermutation types and the numbers of the dinucletitide sequences, GG>AG (red) and GA>AA (blue), were [file pone.0092861.s002.pdf]

A

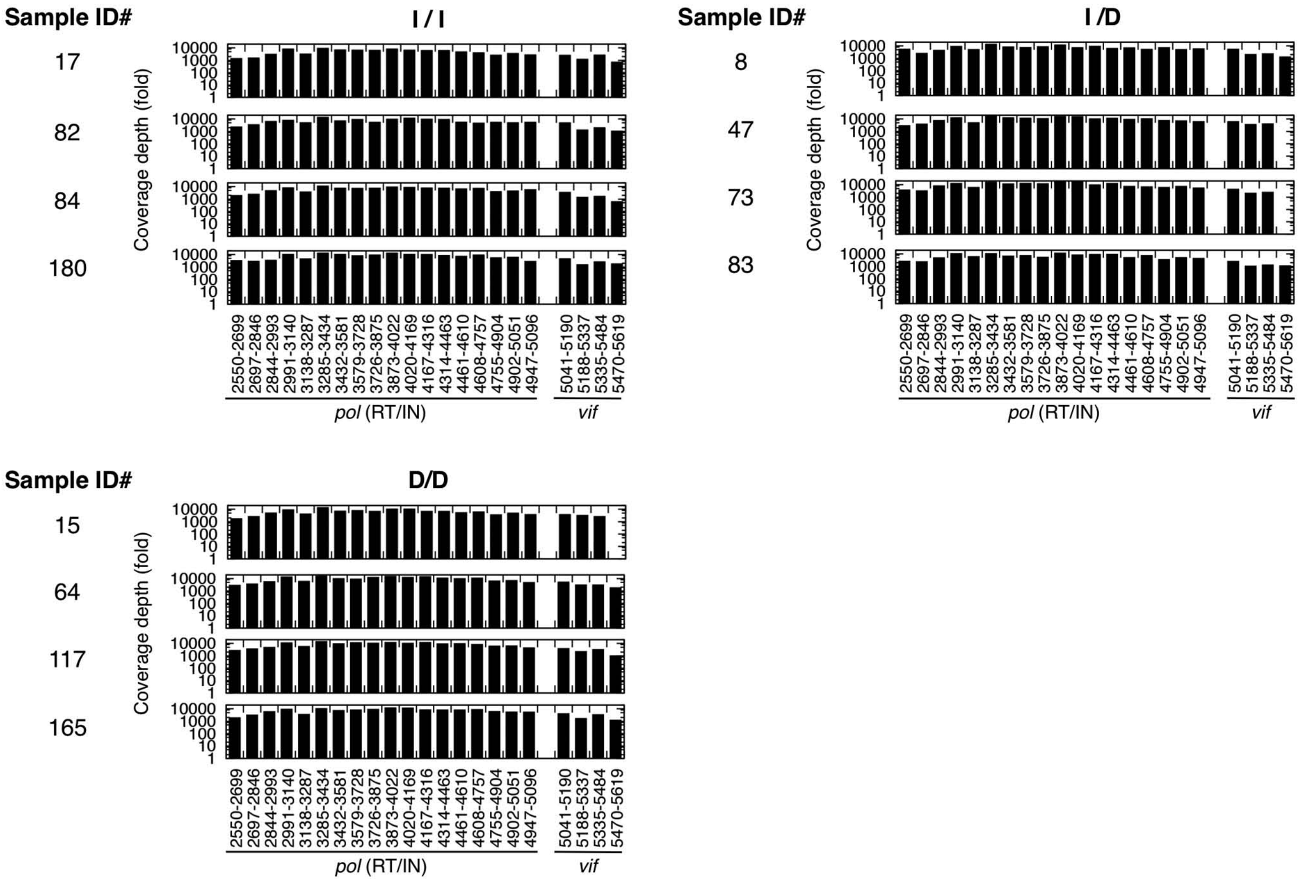

Sample ID#
I / I
17
82
84
180
Coverage depth (fold)
10000
1000
100
10
1
2550-2699
2697-2846
2844-2993
2991-3140
3138-3287
3285-3434
3432-3581
3579-3728
3726-3875
3873-4022
4020-4169
4167-4316
4314-4463
4461-4610
4608-4757
4755-4904
4902-5051
4947-5096
5041-5190
5188-5337
5335-5484
5470-5619
pol (RT/IN)
vif
Sample ID#
I /D
8
47
73
83
Coverage depth (fold)
pol (RT/IN)
vif
Sample ID#
D/D
15
64
117
165
Coverage depth (fold)
pol (RT/IN)
vif

B

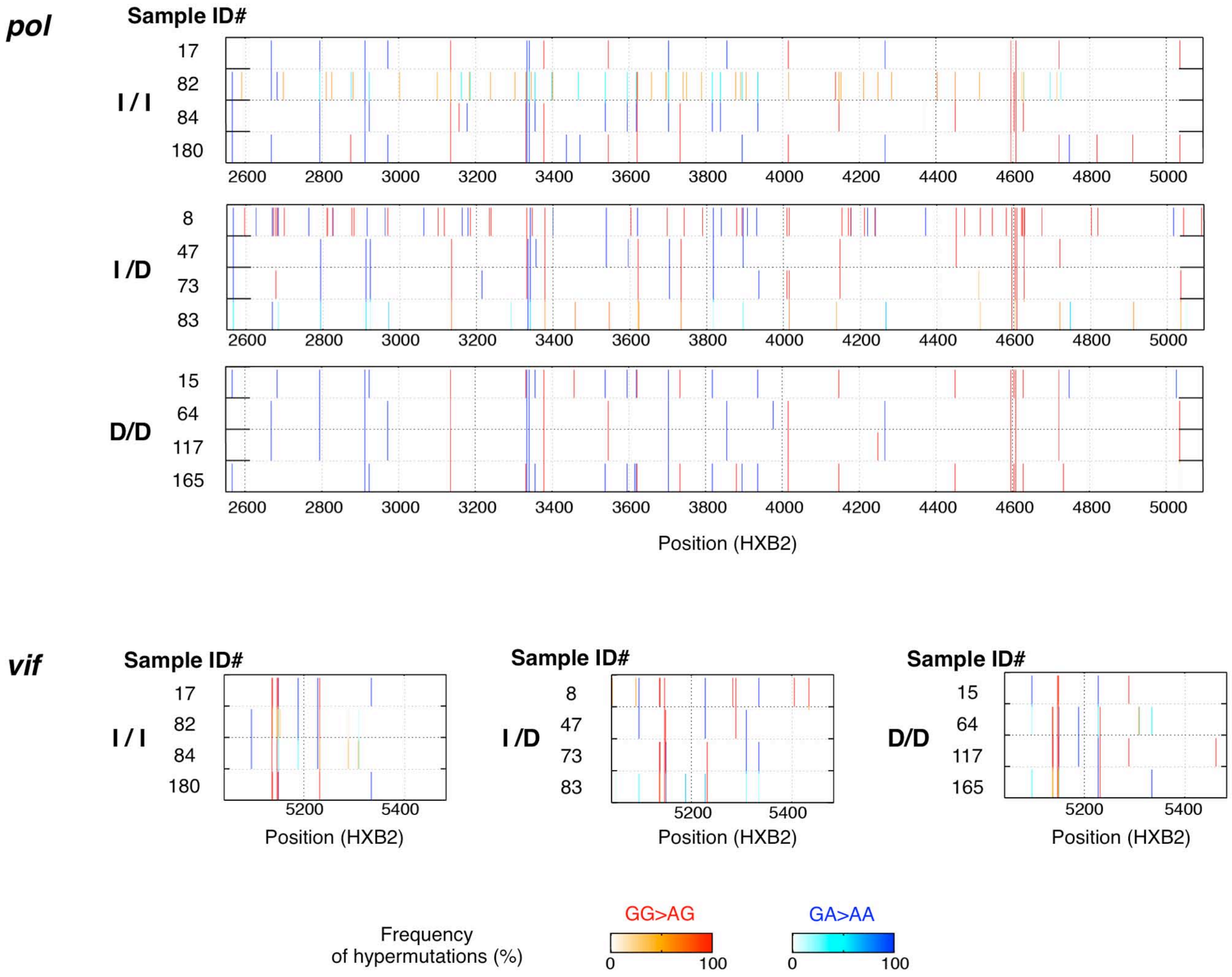

pol
Sample ID#
I / I
17
82
84
180
2600
2800
3000
3200
3400
3600
3800
4000
4200
4400
4600
4800
5000
I /D
8
47
73
83
D/D
15
64
117
165
Position (HXB2)
vif
Sample ID#
5200
5400
Position (HXB2)
GG>AG
GA>AA
Frequency
of hypermutations (%)
0
100

C

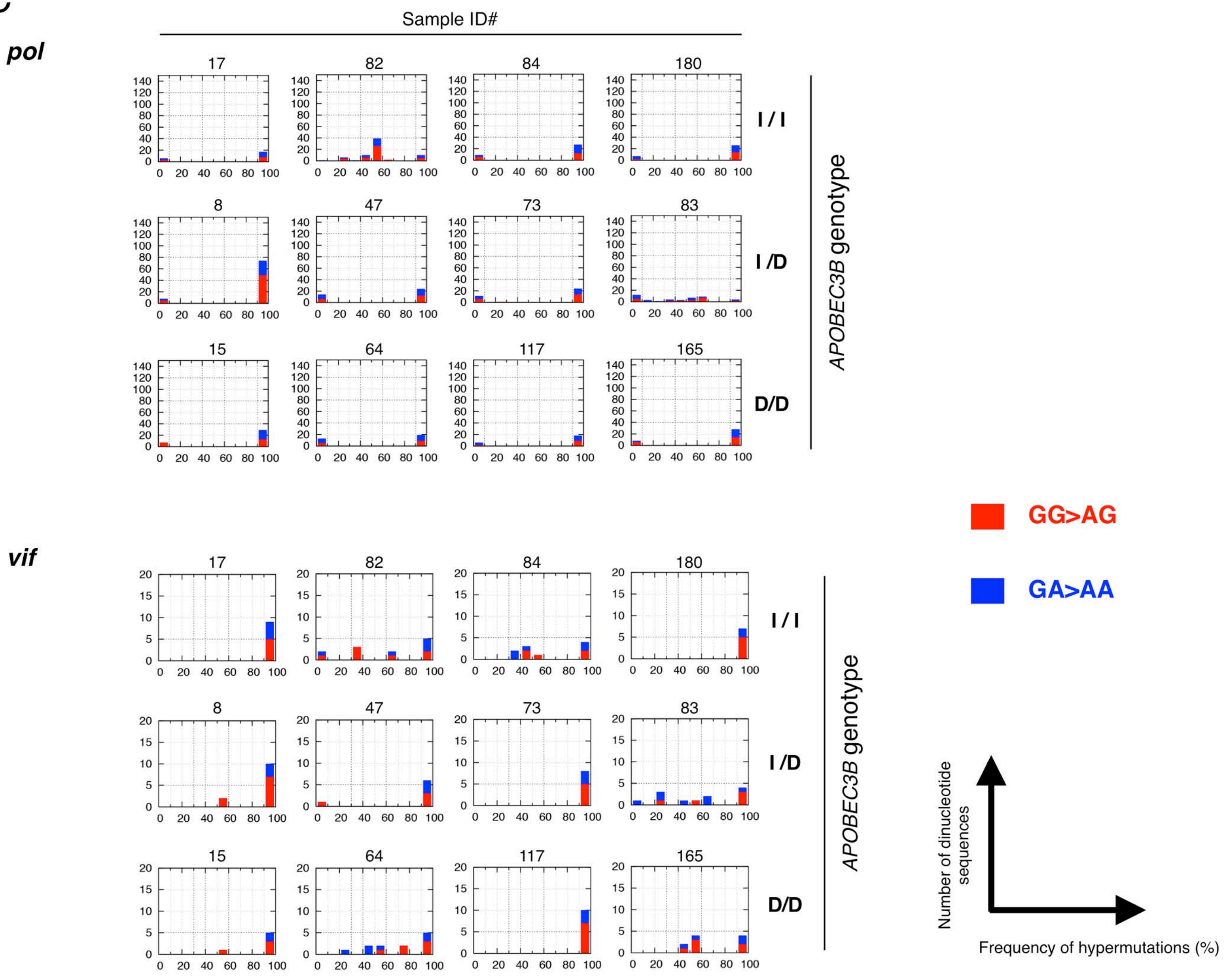
Sample ID#
pol
vif
17
82
84
180
8
47
73
83
15
64
117
165
I / I
I /D
D/D
APOBEC3B genotype
GG>AG
GA>AA
Number of dinucleotide sequences
Frequency of hypermutations (%)
