# Supplementary material for: Lack of Association between Intact/Deletion Polymorphisms of the APOBEC3B Gene and HIV-1 Risk
Source: PLoS One. 2014 Mar 25;9(3):e92861. doi: 10.1371/journal.pone.0092861 (PMC3965477; doi:10.1371/journal.pone.0092861)
Supplement: Table S1 — Oligonucleotide primers used for real-time PCR of APOBEC3 and control. A real-time PCR assay was performed for APOBEC3 and control genes (Gene symbol) using each of forward primer (S) and reverse primer (AS) sets. (DOC) [file pone.0092861.s004.doc]

**Supplementary Data- Table S1**

Oligonucleotide primers for real-time PCR of *APOBEC3*

|  | | | | |
| --- | --- | --- | --- | --- |
| Gene symbol | 5’ Primer Name | Nucleotide Sequences (5’ to 3’) | 3’ Primer Name | Nucleotide Sequences (5’ to 3’) |
| *APOBEC3A* | APOBEC-3A-S-Y | GAAGCCAGCCCAGCATCC | APOBEC-3A-AS-Y | CCCCTGTGCTGGTCCATCTT |
| *APOBEC3B* | APOBEC-3B-S | GAATTCCTGTCTGAGCACCCC | APOBEC-3B-AS | GAGCGCCCTTCGGTAATCTC |
| *APOBEC3C* | APOBEC-3C-S | GGAACGAAACTTGGCTGTGC | APOBEC-3C-AS | CAGAATCCACCTGGTTTCGG |
| *APOBEC3DE* | APOBEC-3D-S | GCTGAGCACCCCAATGTCA | APOBEC-3D-AS | CACCCACCGCCAATCTCT |
| *APOBEC3F* | APOBEC-3F-S | TTCGAGGCCAGGTGTATTCC | APOBEC-3F-AS | GGCAGCTGGTTGCCACAGA |
| *APOBEC3G* | APOBEC-3G-S | GGTCAGAGGACGGCATGAGA | APOBEC-3G-AS | GCAGGACCCAGGTGTCATTG |
| *APOBEC3H* | APOBEC-3H-S-Y | CTGTACTACCACTGGTGCAAGC | APOBEC-3H-AS-Y | TAGGGGTTGAAGGAAAGCG |
| *β-actin* | Actin-S | ATTGCCGACAGGATGCAGAA | Actin-AS | ACATCTGCTGGAAGGTGGACAG |
| *GAPDH* | GAPHD-S | GAAGGTGAAGGTCGGAGTC | GAPHD-AS | GAAGATGGTGATGGGATTTC |
