# Supplementary material for: Lack of Association between Intact/Deletion Polymorphisms of the APOBEC3B Gene and HIV-1 Risk
Source: PLoS One. 2014 Mar 25;9(3):e92861. doi: 10.1371/journal.pone.0092861 (PMC3965477; doi:10.1371/journal.pone.0092861)
Supplement: Table S2 — APOBEC3B variations in the I/D and I/I genotyped healthy donors. APOBEC3B cDNAs from I/D and I/I-genotyped healthy donors (n = 5 each) were amplified by the nested RT-PCR and cloned into pUC118 plasmids. The APOBEC3B cDNA sequences were determined by DNA sequencing. The individual APOBEC3B variants analyzed are shown. A3B, APOBEC3B. (DOC) [file pone.0092861.s005.doc]

| **Table S2**  **APOBEC3B variations in the I /D and I / I genotyped healthy donors** | | | | | |
| --- | --- | --- | --- | --- | --- |
|  | | | | | |
|  | I /D Genotypes | | | | |
| Donor # | 1 | 2 | 3 | 4 | 5 |
| A3B Variations | E62/- | K62/- | K62/- | K62/- | E62/- |
|  | | | | | |
|  | I / I Genotypes | | | | |
| Donor # | 6 | 7 | 8 | 9 | 10 |
| A3B Variations | E62/E62 | E62/K62 | E62/E62 | K62/K62 | E62/E62 |

* APOBEC3B cDNAs from I /D and I / I-genotyped healthy donors (n = 5 each) were amplified by the nested RT-PCR and cloned into pUC118 plasmids. The *APOBEC3B* cDNA sequences were determined by DNA sequencing. The individual APOBEC3B variants analyzed are shown. A3B, APOBEC3B.
